# Effects of a subject‐led social participation program to improve social well‐being of senior citizens in the community

**DOI:** 10.1002/alz.095084

**Published:** 2025-01-09

**Authors:** Inhye Kim, Hyunseo An, Sohyeon Yun, Jiwon Shin, Hyun Yang, Hae Yean Park

**Affiliations:** ^1^ Graduate School, Yonsei University, Wonju, Wonju Korea, Republic of (South); ^2^ College of Software and Digital Healthcare Convergence, Yonsei University, Wonju, Heungup‐meon Korea, Republic of (South)

## Abstract

**Background:**

As populations age, fostering the social participation of older adults is crucial to addressing issues like isolation. This study introduced a subject‐led social participation program to encourage independent engagement among older adults, utilizing a custom‐designed menu of activities. The program’s impact on social well‐being was assessed to explore its practical application and identify potential improvements.

**Method:**

The program was evaluated using a single‐group pre‐post design from February to April 2024. Participants included seniors aged 65 or older from small to medium cities, engaging in social activities less than three times a week, with independent mobility and normal cognitive function. Twelve sessions, each lasting 60‐90 minutes, were conducted over six weeks. Social well‐being improvements were measured using various scales, including SPF‐IL, the Quality of Life Scale for the Elderly, and the UCLA Loneliness Scale 3rd edition. Program activities were categorized into social, rules, and honor activities, each involving planning and implementation phases based on discussions and using the social participation menu.

**Results:**

Four seniors participated in the study. The program positively changed social well‐being, self‐efficacy, and loneliness. Post‐program interviews aligned subjective experiences with observed improvements, indicating effective social integration and continued participation benefits.

**Conclusion:**

The subject‐led program effectively enhances social well‐being, reduces loneliness, and increases self‐efficacy among older adults. Participants reported long‐lasting learning effects and a newfound sense of societal integration, suggesting the program could effectively engage older adults in traditionally perceived as youth‐oriented meaningful activities. However, the small sample size calls for further studies with more participants and a control group to confirm these findings and ensure broader applicability.

